# Fine needle assisted puncture positioning technique for CT-guided percutaneous interventions

**DOI:** 10.3389/fonc.2026.1715384

**Published:** 2026-06-12

**Authors:** Qingqing Wang, Huanjing Wang, Jian Zhang, Zhongbao Tan

**Affiliations:** Department of Interventional Radiology, The Affiliated Hospital of Jiangsu University, Jiangsu University, Zhenjiang, China

**Keywords:** challenging target locations, CT-guided, fine-needle-assisted, percutaneous interventions, puncture positioning

## Abstract

**Objective:**

This study evaluated the feasibility and safety of a fine-needle-assisted, two-step puncture technique developed to improve accuracy in CT-guided percutaneous interventions at challenging target locations, compared with conventional approaches.

**Methods:**

In this retrospective study, CT-guided procedures—including biopsy, preoperative localization, microwave ablation, or percutaneous vertebroplasty—were performed on 40 patients with challenging target locations. In the fine-needle-assisted group, a 20/22-gauge initial fine needle was positioned near the target under CT guidance to serve as a guide. The secondary needle —such as a coaxial biopsy needle, microwave antenna, or bone puncture needle—was then introduced along the same trajectory and angle as the fine needle. The conventional approach involved a direct, stepwise needle insertion toward the lesion in 20 patients with challenging target locations. The number of repositioning attempts for the coaxial needle, microwave ablation antenna, and bone puncture needle, as well as the puncture time, were compared between the two groups. Puncture success rates and complications were recorded.

**Results:**

In the fine-needle-assisted group, initial fine-needle placement was successful in all 20 cases, allowing subsequent puncture of the target lesions. In the conventional approach group, one puncture failure was recorded. No major complications—such as massive pneumothorax, significant hemorrhage, or injury to major vessels or nerves—occurred in either group. The fine needle assisted group required fewer repositioning attempts and shorter puncture times than the conventional approach group, 0.9 ± 0.7 vs. 2.2 ± 1.3 times(p < 0.05) and 12.05 ± 2.86 vs. 14.95 ± 3.32 minutes(p < 0.05), respectively.

**Conclusion:**

The fine-needle-assisted puncture technique offers a safe, feasible, and effective means of improving precision in CT-guided percutaneous procedures at challenging target locations compared with conventional approaches.

## Introduction

In recent years, advances in minimally invasive technology have led to increasing clinical adoption of CT-guided percutaneous procedures (1–4). Although CT-guided percutaneous procedures are recognized as safe and relatively noninvasive diagnostic techniques, they are inevitably associated with a risk of procedure-related complications, such as pneumothorax, hemoptysis, and bleeding (5–7). The conventional approach involved directly advancing the procedural needle. Nevertheless, at challenging target sites—such as small pulmonary or hepatic nodules, areas adjacent to critical structures, or regions affected by respiratory motion—the need for repeated punctures and repositioning makes the procedure highly dependent on operator experience. Numerous technologies—including robotic, electromagnetic, and laser guidance systems—have been developed to enhance the accuracy of CT-guided percutaneous interventions (8–10). However, many of these systems remain experimental or involve complex workflows that rely on external hardware and software.

This study employed a needle-assisted, CT-guided two-step puncture technique for challenging target locations: initial deployment of a reference needle in adjacent tissue, followed by image-guided puncture of the target lesion using the needle as a reference indicator. Here, we retrospectively reviewed all cases involving challenging target locations at our center and evaluated the puncture performance, complications, and procedure-related details of the needle-assisted, CT-guided two-step puncture technique.

## Materials and methods

### Patients

This retrospective study included 20 patients who underwent needle-assisted CT-guided interventions and 20 patients who received the conventional approach, all with the challenging target locations. The puncture location was defined as “challenging target locations” if it met at least one of the following criteria: (1) pulmonary small nodules ≤3 cm; (2) hepatic nodules ≤3 cm within 1 cm of the diaphragmatic dome; and (3) proximity to critical structures, ≤1 cm from major vessels (e.g., main pulmonary artery, aorta), cardiac, spinal cord. Lesions with relatively low puncture difficulty or uncorrectable coagulation dysfunction were excluded. The study received approval from the Ethics Committee of the Affiliated Hospital of Jiangsu University (KY2024K0503).

### Methods

#### Equipment and materials

The equipment and materials used were as follows: Fine needle: a 20 cm long, 20/22 gauge PTC puncture needle (Hakko, Japan). Coaxial technique: a 17 G × 7 cm Coaxial Introducer Needle and an 18 G × 10 cm BioPince™ Full Core Biopsy Instrument (both from Argon Medical Devices, Inc., USA). Preoperative localization needle: sourced from Ningbo Shengjie Kang Biotechnology Co., Ltd. (Ningbo, China). Microwave ablation (MWA) antenna: sourced from Nanjing ECO Medical Equipment Co., Ltd. (Nanjing, China). Bone puncture needle: a 13 gauge × 130 mm device from Shandong Guanlong Medical Utensils Co., Ltd. (Jinan, China). Polymethylmethacrylate bone cement: Osteopal V (Heraeus, Germany).

#### Operation

Prior to the puncture procedure, the patient was positioned appropriately (lateral, supine, or prone) based on preoperative radiological findings. The needle-assisted CT-guided intervention was performed as follows: (1) CT scanning was first performed to plan the needle insertion path, including the skin puncture site, needle angle, and insertion depth. (2) Procedures such as percutaneous biopsy (lung or liver), preoperative localization, and percutaneous vertebroplasty (PVP) were performed under local anesthesia, whereas microwave ablation was performed under monitored anesthesia care. (3) Under CT guidance, the fine needle was advanced along the predetermined trajectory to the edge of the target lesion. (4) Using the position and angle of this needle, along with the preplanned CT parameters, a second puncture was performed. (5) The coaxial needle was advanced toward the pulmonary or hepatic lesion along the trajectory and angle established by the fine needle. A confirmatory CT scan confirmed that the needle tip had reached the target depth within the lesion. The biopsy needle was then inserted through the coaxial needle to rapidly sample the tissue. Finally, both the fine needle and the coaxial needle were removed. (6) Under CT guidance, the Hook-wire needle was advanced along the fine needle’s path to the predetermined depth and then deployed. (7) The microwave ablation (MWA) antenna, measuring 1.6 mm or 1.8 mm in diameter, was inserted into the pulmonary or hepatic lesion along the trajectory of the fine needle. For pulmonary lesions, the MWA power was set to 30–50 W and applied for 2–5 minutes. Following pulmonary ablation, the MWA antenna was gradually withdrawn while performing parallel needle tract ablation at 20 W for 1 minute. Finally, the fine needle was removed. For hepatic lesions, the MWA power was set to 50–70 W and applied for 5–10 minutes. In these cases, the fine needle could be removed prior to ablation. After hepatic ablation, the MWA antenna was gradually withdrawn with parallel needle tract ablation, again at 20 W for 1 minute. (8) The bone puncture needle was inserted into the vertebral body along the trajectory of the fine needle, which was then removed. A unilateral pedicle puncture was used if the tumor was confined to one side of the vertebral body without crossing the midline; otherwise, a bilateral approach was taken. The MWA antenna was advanced coaxially along the bone puncture needle to the tumor’s anterior edge. After ablation, the antenna was withdrawn, and the bone puncture needle was re-advanced to the same margin. Polymethylmethacrylate bone cement was injected (0.3–0.5 mL per dose), with CT performed after each injection to assess distribution and leakage. (9) After the procedure, a non-contrast CT scan was performed to detect pneumothorax, bleeding, or other complications. In the conventional approach group, the procedural needle was advanced directly, with no prior fine needle placement.

### Outcome assessment

The success rate for puncturing the target lesion was recorded separately for the fine needle, coaxial needle, biopsy needle, MWA antenna, and bone puncture needle. Technical success of the fine needle was defined as providing an accurate reference for subsequent puncture without causing complications. Technical success for biopsy was defined as acquisition of sufficient tissue for pathological analysis; for MWA, as accurate targeting of the nodule. Puncture time was defined from the first CT scan for path planning to final needle position confirmation. The number of needle (coaxial needle, MWA antenna, and bone puncture needle) repositioning attempts was collected. Complications related to the puncture procedure, including pneumothorax and bleeding, were also collected.

### Statistical analysis

All data were analyzed using SPSS 26.0 (SPSS, Chicago, IL, USA). Continuous variables are presented as mean ± standard deviation, and categorical variables as frequencies and percentages. Between-group comparisons of categorical data employed the chi-square test or Fisher’s exact test. For continuous data, differences were evaluated using Student’s t-test or the Mann–Whitney U test. A *p* value < 0.05 was considered statistically significant.

## Results

**Figure 1:**
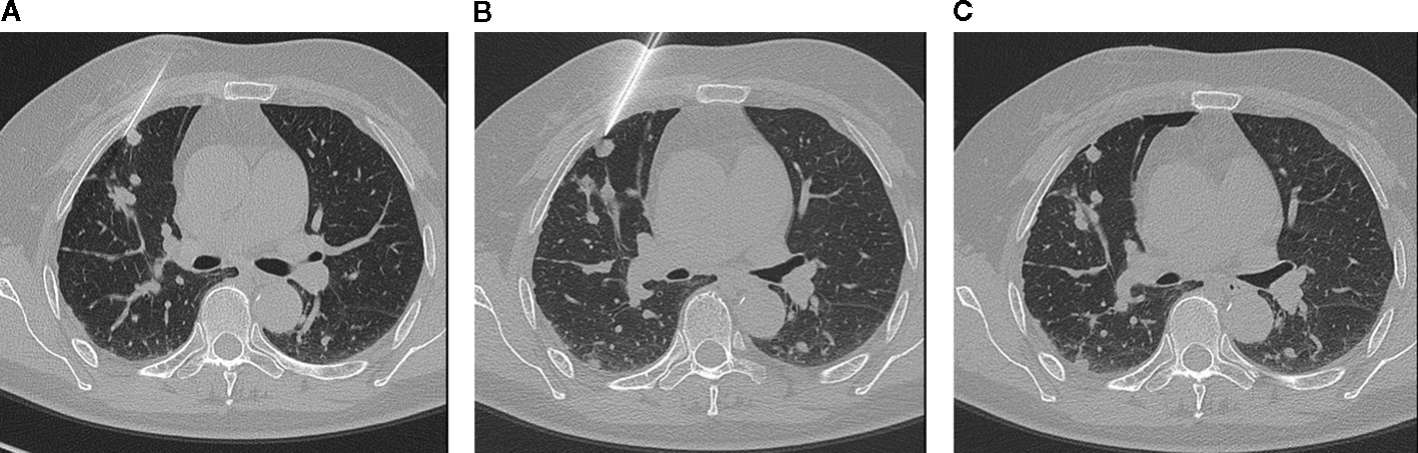
Fine needle-assisted puncture positioning technique in computed tomography-guided percutaneous biopsy. **(A)** A 22 G PTC fine needle was inserted near the tumor nodules. **(B)** A coaxial puncture needle was gradually inserted near the tumor guided by the mark of the fine needle. **(C)** CT scan showed slight pneumothorax.

**Figure 2:**
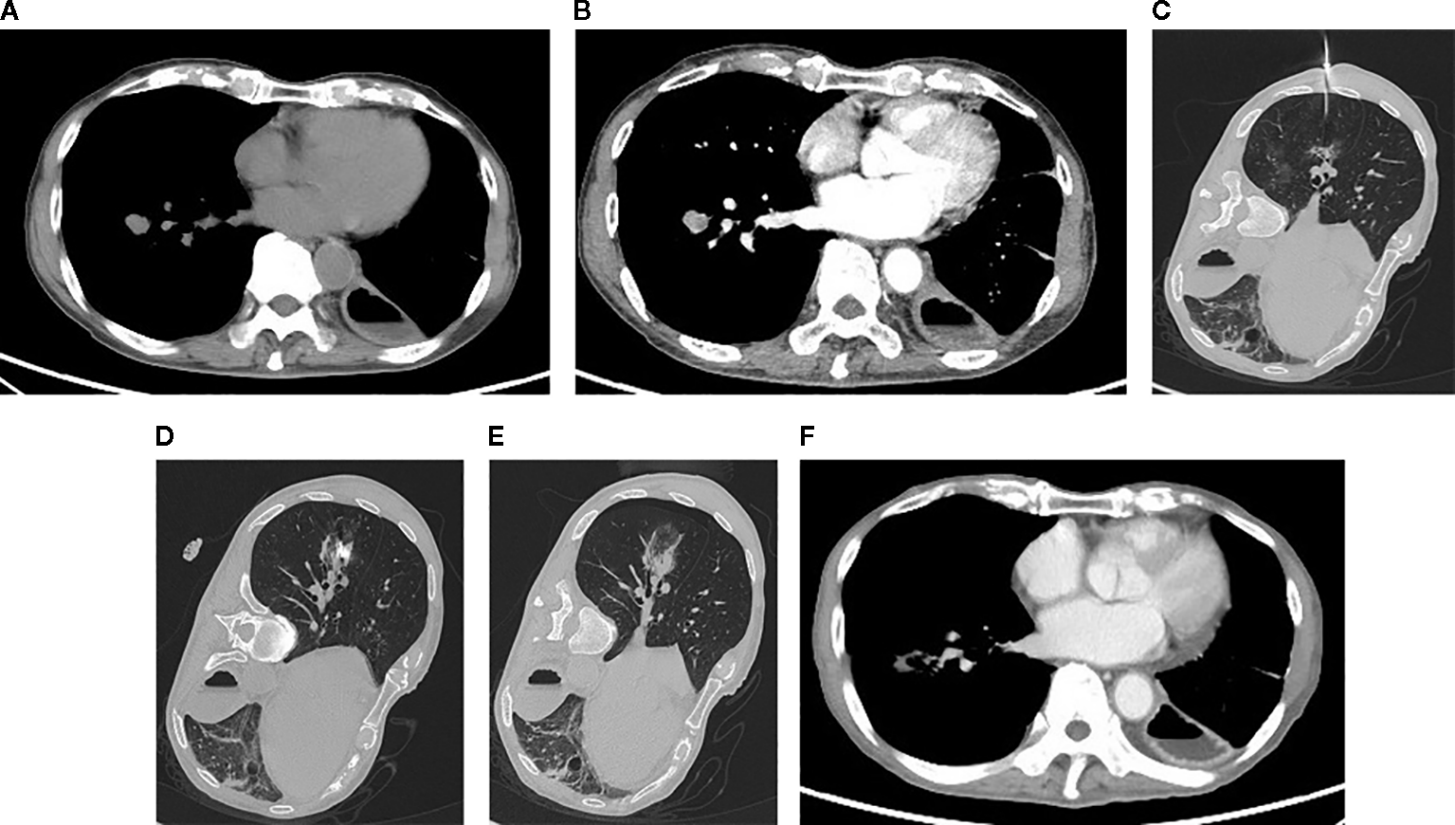
Fine needle-assisted puncture positioning technique in computed tomography-guided microwave ablation for a patient with small cell lung cancer. **(A, B)** Enhanced CT scans showed a tumor mass near the right hilum. **(C)** A 22-gauge PTC fine needle was inserted near the tumor mass. **(D)** A microwave electrode needle was gradually inserted into the tumor according to the mark of the fine needle while the patient held their breath. **(E)** CT scan showed no serious complications. **(F)** Stable disease was observed on CT scan 1 year post-microwave ablation.

**Figure 3:**
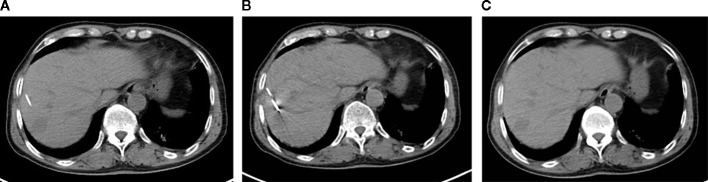
Fine needle-assisted puncture positioning technique in computed tomography-guided percutaneous biopsy. **(A)** A 22-gauge PTC fine needle was inserted near the tumor nodules. **(B)** A coaxial puncture needle was gradually inserted near the tumor according to the mark of the fine needle. **(C)** CT scan showed no hemorrhage.

**Figure 4:**
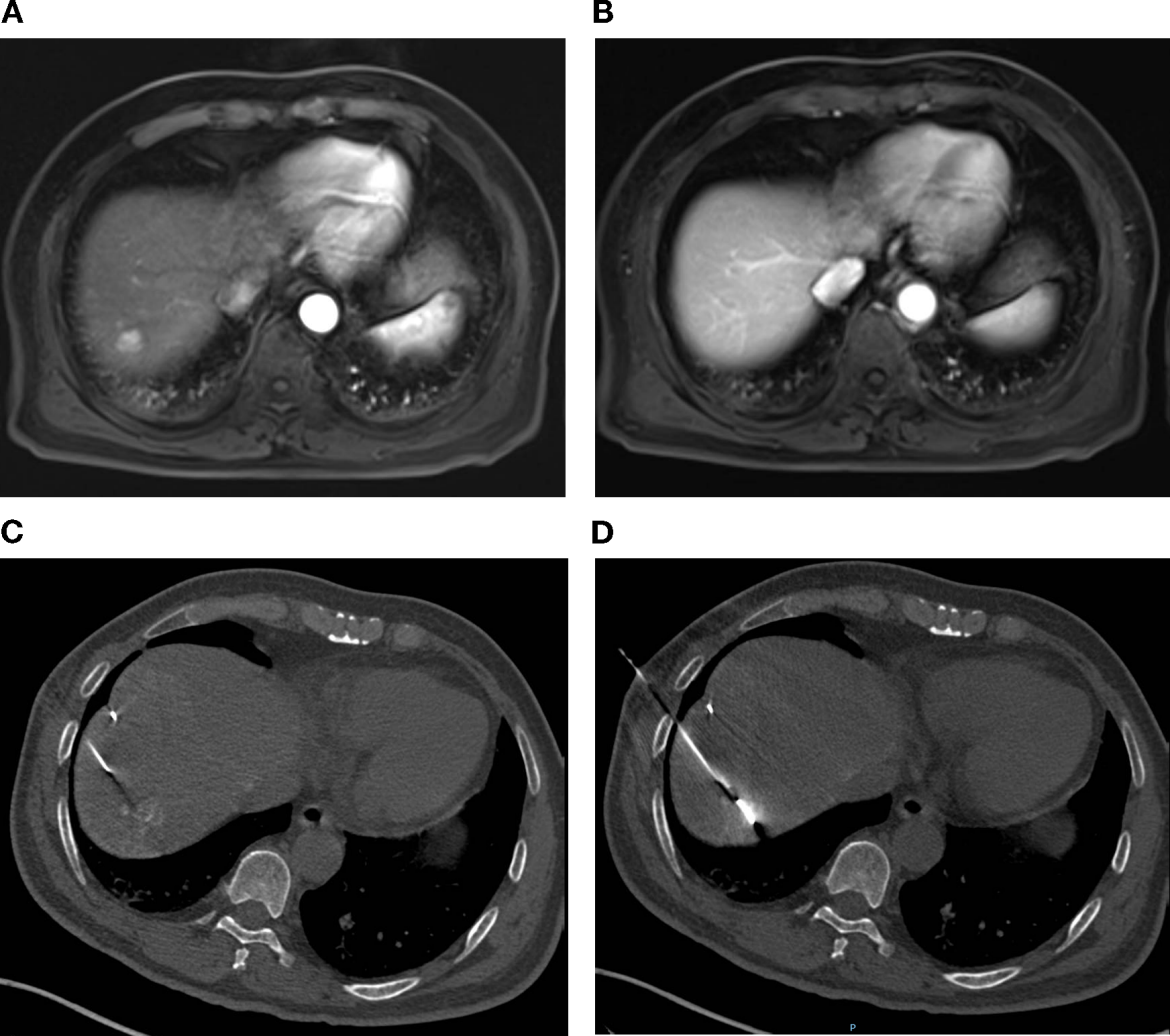
Fine needle-assisted puncture positioning technique in computed tomography-guided microwave ablation for a patient with hepatocellular carcinoma in liver segment S7. **(A, B)** enhanced MRI scans showed a tumor mass in liver segment S7. **(C)** a 22-gauge PTC fine needle was inserted near the tumor mass. **(D)** a microwave electrode needle was gradually inserted into the tumor according to the mark of the fine needle while the patient held their breath.

**Figure 5:**
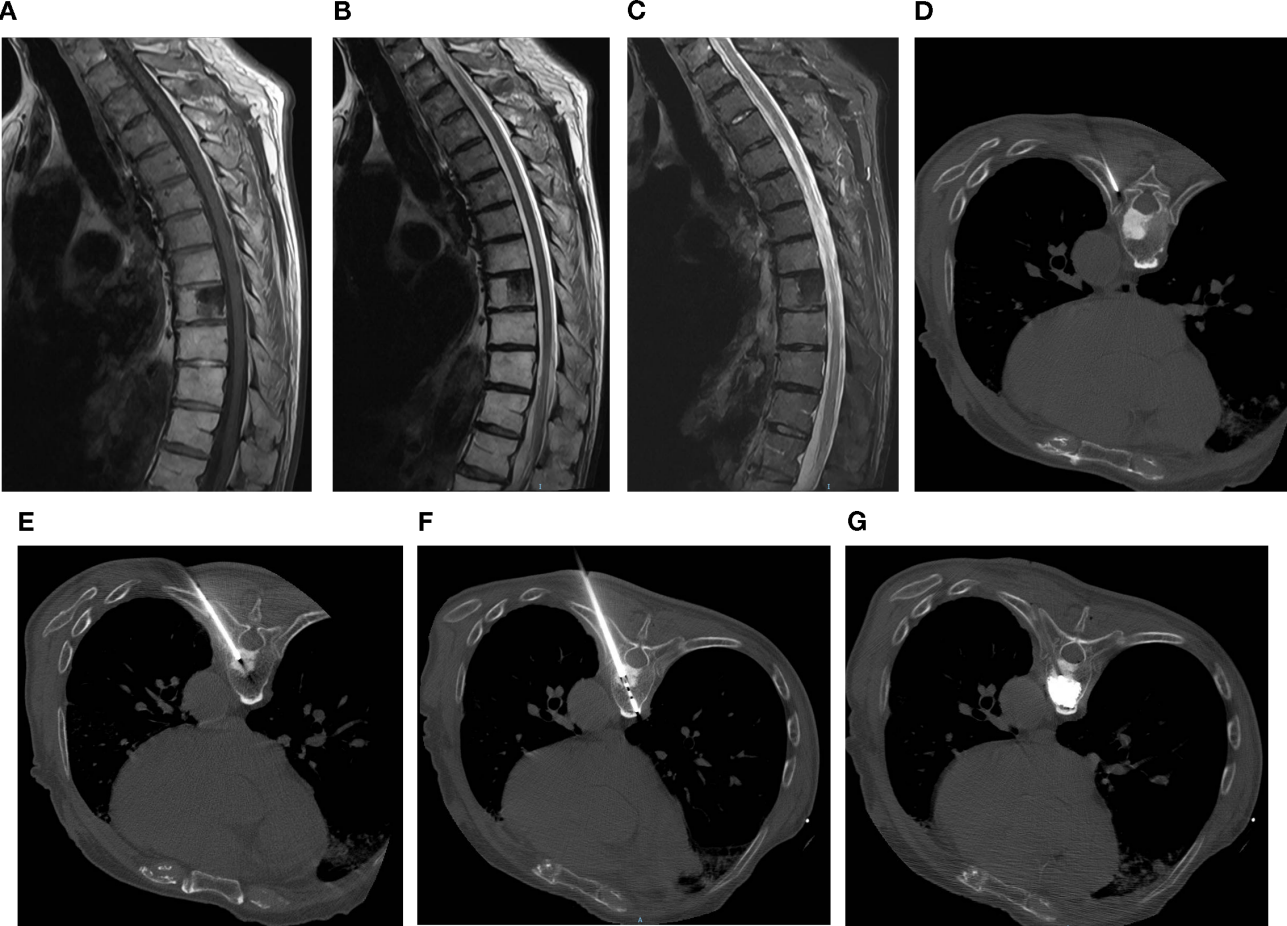
Fine needle-assisted puncture positioning was employed during computed tomography-guided percutaneous vertebroplasty combined with microwave ablation in a patient with lung cancer metastatic to the sixth thoracic vertebral body. **(A–C)** MRI scans showed a tumor mass in the sixth thoracic vertebral body. **(D)** A 20-gauge PTC fine needle was inserted near the tumor mass. **(E)** A bone puncture needle was gradually inserted into the tumor according to the mark of the fine needle. **(F)** CT scan showed that the microwave ablation needle was located in the tumor body. **(G)** bone cement (3 mL) was injected after ablation (20 W, 2 minutes).

Among the needle-assisted group, all patients underwent puncture positioning with a 20/22G fine needle, which served as an auxiliary needle for localization. Under CT guidance, these lesions were successfully localized by placing the needle at the lesion’s edge, 1 cm from the lesion, or along the puncture path. In the needle-assisted group, the success rate for fine-needle-assisted localization was 100% (**Figures 1**–**5**), and the success rate for biopsy or microwave ablation was also 100%. In the conventional approach group, biopsy failed in one patient owing to significant respiratory motion, preventing the acquisition of adequate material for pathological analysis. Compared with the conventional approaches group, the needle-assisted group required fewer repositioning attempts for the coaxial needle, MWA antenna, and bone puncture needle (0.9 ± 0.7 vs. 2.2 ± 1.3, p < 0.001) and had a significantly shorter mean puncture time (12.05 ± 2.86 vs. 14.95 ± 3.32 minutes, p =0.005) (**Table 1**).

**Table 1:** Patients baseline and puncture-related data.

Variable	Needle-assisted group (n=20)	Conventional approaches group (n=20)	p
Sex			0.749^a^
Female	8 (40%)	9 (45%)	
Male	12 (60%)	11 (55%)	
Age (years)	64.6 ± 14.8	61.3 ± 14.9	0.401^b^
Nodule location			0.342^c^
Lung	16 (80%)	19 (95%)	
Liver	3 (15%)	1 (5%)	
Spine	1 (5%)	0 (0%)	
Procedure			0.626^a^
Biopsy	7 (35%)	5 (25%)	
Lung nodule localization	5 (25%)	8 (40%)	
MWA	8 (40%)	7 (35%)	
Technical success (%)	20 (100%)	19 (95%)	1.000^c^
Puncture time (min)	12.05 ± 2.86	14.95 ± 3.32	0.005^d^
Needle repositioning(times)	0.9 ± 0.7	2.2 ± 1.3	<0.001^b^

Data for variable “a” were compared using the Chi-square test, “b” using the Mann-Whitney U test, “c” using Fisher’s exact test, and “d” using Student’s t test.

The initial fine needle was inserted to reach the edge of the target lesion without any puncture-related complications. No serious complications—such as massive or tension pneumothorax, liver rupture with hemorrhage, or injuries to nerves, the spinal cord, or major blood vessels—were observed in either group.

## Discussion

CT-guided punctures are widely used in clinical practice because of their high spatial and density resolution. However, targeting lesions in challenging locations—such as small nodules, those affected by significant respiratory motion, or lesions abutting critical organs—carries a relatively high risk of requiring multiple needle adjustments. Such adjustments are well-recognized risk factors for adverse events. In this retrospective study, we compared a 20–22 G fine-needle-assisted two-step puncture technique with a conventional direct puncture approach in patients with challenging target locations. The fine-needle-assisted technique significantly reduced the number of needle repositioning attempts (0.9 ± 0.7 vs. 2.2 ± 1.3, p < 0.001) and shortened the mean puncture time (12.05 ± 2.86 vs. 14.95 ± 3.32 minutes, p = 0.005), while maintaining a high technical success rate (100% vs. 95%). No major complications occurred in either group. These findings indicate that the fine-needle-assisted technique is a feasible, safe, and effective approach for CT-guided percutaneous procedures in the challenging target locations.

Tsai SC et al. (11) employed a laser to guide pulmonary nodule localization to improve puncture accuracy. Similarly, Weigel et al. (10) used a 3D laser guidance system for needle placement in an abdominal phantom. These techniques improve the accuracy of needle angle placement *in vitro*. A robotic assistance system (RAS) has also been applied to place needles into fixed lesions (8, 9). However, CT-guided punctures of the lung and liver are susceptible to patient respiratory motion, which can increase complication rates—particularly for small lesions, those located in the lower lungs, or liver nodules adjacent to the diaphragm (12). Park et al. (13) suggested that performing pleural puncture during the expiratory phase may reduce the risk of pneumothorax in image-guided lung biopsy. In their study, the maximal respiratory motion of nodules was 5.5 ± 4.4 mm (range 0–40 mm). For beginners, pleural puncture becomes especially difficult when respiratory motion substantially shifts the puncture site, markedly increasing the risk of failure. Wu et al. (14) demonstrated that fine-needle-mediated breathing control could be applied during CT-guided percutaneous puncture of small lung or liver nodules adjacent to the diaphragm, thereby improving success rates and reducing complications. In their study, a 23G fine needle was first positioned vertically near the planned puncture path to simulate respiratory motion. During the breath-hold phase, the needle was inserted in this vertical orientation, aligned parallel to the X-ray beam. This configuration minimized the needle’s radiographic visibility throughout the procedure. Niebur et al. (7) identified the distance from the liver capsule to the lesion as a significant predictor of post-biopsy hemorrhage. This capsular-to-lesion path length directly affects the number of needle adjustments needed. Reducing such corrections therefore lowers the risk of complications.

In our investigation, a 20–22 G fine needle was inserted and advanced under CT guidance during normal breathing according to predetermined parameters, with its angle and direction closely matching those intended for the subsequent puncture instrument. This approach offers several advantages: (1) Minimally invasive fine needle insertion is safe and does not compromise subsequent procedures. (2) The second puncture can be performed by replicating or minimally adjusting the angle and direction based on the fine needle. (3) It reduces the operator’s reliance on experience, particularly for the challenging target locations that typically demand considerable skill to determine the optimal entry point and trajectory. (4)Using the fine needle as a marker, the second puncture is unaffected by respiratory motion during breath-holding and requires fewer needle repositioning attempts. (5) Using the fine needle as a guide, coaxial or microwave puncture needles can bypass the costophrenic angle when accessing the liver, thereby avoiding lung injury. This approach prevents the needle from penetrating lung tissue via the subcostal margin and reduces the risk of damage to other vital organs. (6) CT-guided puncture does not provide real-time visualization, which can render targeting of high-risk locations unsafe; in contrast, fine-needle assistance offers near real-time guidance and enhances procedural safety. In this study, we recommend that when a fine needle traverses the lung for auxiliary positioning, it should be withdrawn only after the procedure is complete to avoid premature removal, which could cause pneumothorax and interfere with subsequent operations. In other cases, the needle may be removed immediately after the second puncture is successfully performed.

Our study had several limitations. First, variability in CT scanning parameters and anatomical regions precluded a comparison of radiation dose between the two groups. Second, as a single-center retrospective study with a relatively small sample size, selection bias cannot be ruled out. Third, we did not compare the fine-needle-assisted technique with other advanced guidance methods, such as robotic or electromagnetic navigation. Future prospective studies with larger cohorts and direct comparisons to alternative guidance systems are warranted.

## Conclusion

The results of this study suggest that the fine-needle can avoid breathing interference and provide accurate angle and direction for needle placement guidance. The needle-assisted puncture positioning technique in CT-guided percutaneous procedures is safe and feasible.

## Data Availability

The raw data supporting the conclusions of this article will be made available by the authors, without undue reservation.
