# Correction: Fine needle assisted puncture positioning technique for CT-guided percutaneous interventions

**DOI:** 10.3389/fonc.2026.1908614

**Published:** 2026-07-20

**Authors:** Qingqing Wang, Huanjing Wang, Jian Zhang, Zhongbao Tan

**Affiliations:** Department of Interventional Radiology, The Affiliated Hospital of Jiangsu University, Jiangsu University, Zhenjiang, China

**Keywords:** challenging target locations, CT-guided, fine-needle-assisted, percutaneous interventions, puncture positioning

There was a mistake in the caption of **Figures 1–3** as published. The error in the original caption could mislead readers. The corrected caption of figures appears below.

“Figure 1, Fine needle-assisted puncture positioning technique in computed tomography-guided percutaneous biopsy. **(A)** A 22 G PTC fine needle was inserted near the tumor nodules. **(B)** A coaxial puncture needle was gradually inserted near the tumor guided by the mark of the fine needle. **(C)** CT scan showed slight pneumothorax.

Figure 2, Fine needle-assisted puncture positioning technique in computed tomography-guided microwave ablation for a patient with small cell lung cancer. **(A, B)** Enhanced CT scans showed a tumor mass near the right hilum. **(C)** A 22-gauge PTC fine needle was inserted near the tumor mass. **(D)** A microwave electrode needle was gradually inserted into the tumor according to the mark of the fine needle while the patient held their breath. **(E)** CT scan showed no serious complications. **(F)** Stable disease was observed on CT scan 1 year post-microwave ablation.

Figure 3, Fine needle-assisted puncture positioning technique in computed tomography-guided percutaneous biopsy. **(A)** A 22-gauge PTC fine needle was inserted near the tumor nodules. **(B)** A coaxial puncture needle was gradually inserted near the tumor according to the mark of the fine needle. **(C)** CT scan showed no hemorrhage.”

The original version of this article has been updated.

